# Small molecule inhibitors against PD-1/PD-L1 immune checkpoints and current methodologies for their development: a review

**DOI:** 10.1186/s12935-021-01946-4

**Published:** 2021-04-27

**Authors:** Chang Liu, Navindra P. Seeram, Hang Ma

**Affiliations:** Bioactive Botanical Research Laboratory, Department of Biomedical and Pharmaceutical Sciences, College of Pharmacy, University of Rhode Island, Avedisian Hall Lab 440, 7 Greenhouse Road, Kingston, RI 02881 USA

**Keywords:** Cancer, Immunotherapy, PD-1/PD-L1, Natural products, Small molecules

## Abstract

Programmed death-1/programmed death ligand-1 (PD-1/PD-L1) based immunotherapy is a revolutionary cancer therapy with great clinical success. The majority of clinically used PD-1/PD-L1 inhibitors are monoclonal antibodies but their applications are limited due to their poor oral bioavailability and immune-related adverse effects (irAEs). In contrast, several small molecule inhibitors against PD-1/PD-L1 immune checkpoints show promising blockage effects on PD-1/PD-L1 interactions without irAEs. However, proper analytical methods and bioassays are required to effectively screen small molecule derived PD-1/PD-L1 inhibitors. Herein, we summarize the biophysical and biochemical assays currently employed for the measurements of binding capacities, molecular interactions, and blocking effects of small molecule inhibitors on PD-1/PD-L1. In addition, the discovery of natural products based PD-1/PD-L1 antagonists utilizing these screening assays are reviewed. Potential pitfalls for obtaining false leading compounds as PD-1/PD-L1 inhibitors by using certain binding bioassays are also discussed in this review.

## Introduction

Tumors can bypass immune surveillance by exploiting immune-escape mechanisms including the induction of an immunosuppressive microenvironment and suppression of effector T cells’ function in the tumor microenvironment [1, 2]. Cancer immunotherapy is designed to re-activate anti-tumor immune response and enhance its effects, thereby restoring tumor immune suppression [3–5]. Activating T cell-mediated anti-tumor responses is one of the most effective strategies on the basis of the regulation of immune checkpoints, which are crucial receptors for preventing autoimmunity, protecting the host from tissue damage, and regulating self-tolerance [6–8].

T cell-mediated cancer immunotherapy is a breakthrough since its discovery [9, 10]. The activation of cancer-specific T cells eliminates cancer cells by the recognition of tumor-specific antigens [10, 11]. T cell-mediated cancer immunotherapy consists of three steps. First, antigens are presented by antigen-presenting cells (APCs) such as dendritic cells (DCs) as antigenic peptides, which are recognized by the T-cell receptor (TCR; Signal 1) [12]. The secondary signal is then delivered when B7 proteins (CD80 and CD86) on the APCs engage with CD28 on the T cells, leading to the activation of T cells [13]. Subsequently, the activated cancer-specific T cells enter into the tumor sites and recognize tumor-specific antigens thereby destroying the cancer cells [13]. However, in the tumor microenvironment, cancer cells highly express co-inhibitory protein ligands including CD80/86 and programmed death-ligand 1 (PD-L1) [14–16]. Co-inhibitory proteins including cytotoxic T-lymphocyte-associated protein 4 (CLTA-4) and programmed cell death protein 1 (PD-1) are activated by binding to their ligands expressed on cancer cells [17–19]. Consequently, cancer-specific T cell activation is prevented so the cancer cells can escape from immune surveillance. Therefore, blockage of the co-inhibitory signals on the T cells and the activation of cancer-specific T cells represent a promising strategy in cancer immunotherapy.

PD-1 is a co-inhibitory receptor mainly expressed on the surface of T cells [20]. PD-1’s primary function is to suppress the T cells’ activity by the regulation of the TCR signaling cascade [21–23]. High PD-L1 expression in tumor microenvironment is frequently observed in many types of cancers including Hodgkin’s lymphoma, breast cancer, renal cell carcinoma, melanoma, lung cancer, gastric cancer, and hepatoma [24–30]. In the tumor microenvironment, PD-L1 binds to PD-1 leading to T cell dysfunction, whereas blockage of their interactions recovers the T cell’s activity of destroying tumor cells [31, 32]. Previous studies reported that the blockage of the PD-L1/PD-1 interactions is a promising strategy for cancer immunotherapy [18, 32, 33]. Blockage of PD-L1/PD-1 interactions can terminate the PD-1 mediated-signaling pathways and reactivate the T cell-mediated anti-tumor responses by promoting T cell proliferation and enhancing effector T cell’s function [32, 34]. Clinical studies reported that the blockade of PD-1/PD-L1 interactions can boost T cell-mediated antitumor responses, generate durable clinical responses, and prolong patient survival rate [17, 35]. To date, monoclonal antibodies (mAbs) targeting PD-1 (e.g. Cemiplimab, Nivolumab, and Pembrolizumab) or PD-L1 (e.g. Durvalumab, Avelumab, and Atezolizumab) are approved by the United States FDA for the treatment of a series of malignancies [16, 36–38]. Although these mAbs exhibit promising therapeutic effectiveness in clinical studies, restrictions including immune-related adverse effects, immunogenicity, and high costs are imposed for the clinical utilization of antibody-based immune checkpoint inhibitors [15, 17, 39, 40]. In addition, these mAbs exert limited permeability in the tumor tissues due to their large size [41, 42]. Their relatively long half-life increases the difficulty in drug elimination, which may lead to severe side effects. Alternatively, small molecule inhibitors may possess favorable tumor penetration and oral bioavailability [42]. Moreover, small molecule inhibitors may exert other advantages such as fewer side effects, are easier self-administered, have shorter biological half-life, and are less expensive than mAbs, which have attracted great attention in pharmaceutical industries. However, most small molecule inhibitors against PD-1/PD-L1 are still in the early drug development stage with a focus on preclinical studies.

Currently, preclinical studies have demonstrated that small molecule inhibitors can exhibit superior capacities to inhibit tumor growth with favorable biosafety as compared to mAbs [42]. Among these small molecule inhibitors, several synthetic small molecules from Bristol Myers Squibb (e.g. BMS1166 and BMS202) and Curis Inc. (i.e. CA-170) exhibit promising tumor suppression effects in interrupting the PD-1/PD-L1 interactions [43, 44]. However, there are relatively fewer reports and pre-clinical studies on natural product-derived small molecule inhibitors.

Bioassays are crucial to assess the blockage effects of small molecules against the PD-1/PD-L1 interactions as well as their binding affinities and how their biological functions impact PD-1/PD-L1. Currently, bioassays to determine the potency of small molecule inhibitors against PD-1/PD-L1 include biophysical and biochemical assays, in vitro cell-based assays, and in vivo tumor xenograft model [45–47]. Biophysical and biochemical assays are used for the assessment of small molecule binding profiles and for the screening of potential inhibitors. In vitro cell-based assays and in vivo tumor xenograft models can evaluate small molecules’ functional effects on PD-1/PD-L1. In addition, due to the encouraging promise of small molecule inhibitors against PD-1/PD-L1, researchers have developed various robust and effective assays for screening PD-1/PD-L1 inhibitors.

Herein, PD-1/PD-L1 immune checkpoints and their interactions are summarized. In addition, natural product-based small molecule inhibitors against PD-1/PD-L1 and current methodologies employed for their development are reviewed. The potential pitfalls and future of small molecule inhibitors against PD-1/PD-L1 are also examined.

## PD-1/PD-L1 and their interactions

PD-1 (CD279) is a transmembrane protein consisting of 288 amino acids belonging to the CD28 superfamily [28]. The structure of PD-1 consists of an extracellular IgV domain connected to a transmembrane region and an intracellular tail, which contains two phosphorylation sites on two motifs including the immunoreceptor tyrosine-based switch motif (ITSM) and immunoreceptor tyrosine-based inhibitory motif (ITIM) [28]. Immunoglobulin (Ig)-like extracellular domain is responsible for engagement and signaling transduction to intracellular domain. After engagement with PD-L1 (CD274; B7-H1) and PD-L2 (CD273; B7-DC), PD-1 delivers ‘negative’ signals to T cells to suppress T cell’s activity. In addition, PD-1 is expressed on the surface of regulatory T cells, activated B cells, monocytes, macrophages, DCs, and natural killer cells [48]. However, the mechanisms of the regulation of PD-1 signaling pathways on these cells are unclear.

PD-1 expression is dynamically changed and intricately regulated by host immune responses [49, 50]. Usually, it is expressed at a low, basal level in resting naive T cells (Th0 cells) to maintain immunological tolerance. However, PD-1 is upregulated by a series of immune cells including CD4 and CD8 T cells, B cells, macrophages, and DCs in response to initial immune stimuli [51]. PD-1 is often down-regulated when the antigen is eliminated but its down-regulation can be observed prior to antigen clearance in the case of acute antigen exposure. By contrast, PD-1 expression maintains a high level in chronically stimulated antigen-specific T cells, which leads to their functional exhaustion in response to stimuli [52].

Similar to other B7 proteins, PD-L1 and PD-L2 are transmembrane glycoproteins [53]. Compared to PD-L2, PD-L1 is expressed on a variety of normal and immune cells including macrophages and DCs as well as cancer cells after exposure to pro-inflammatory stimuli [31]. In addition, PD-L2 is inducibly expressed in hematopoietic cells including macrophages, DCs, mast cells, and certain B cell populations [54, 55]. In the tumor microenvironment, PD-L1 expressed by cancer cells binds to its receptor PD-1 located on activated T cells on the tumor sites. This interaction consequently triggers inhibitory signals to the T cells and prevents the host immune system from suppressing the growth of tumor [56].

**Fig. 1:**
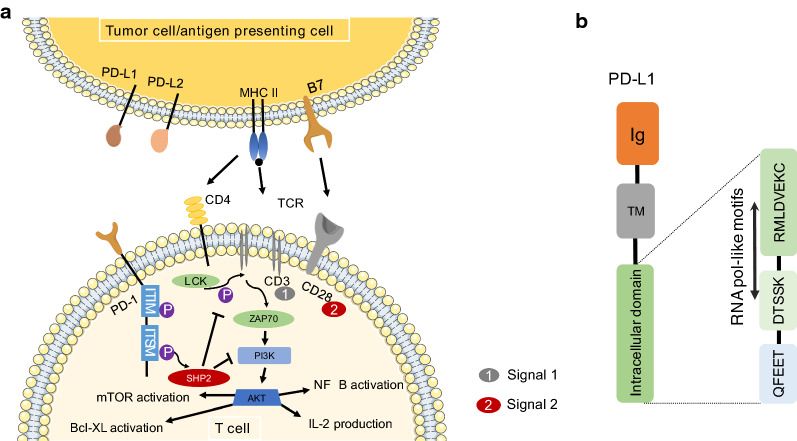
The signaling pathway of PD-1/PD-L1. **a** PD-L1 consists of an extracellular domain, a transmembrane domain, and an intracytoplasmic region but lacks intracellular signaling. The intracytoplasmic region consists of three conserved sequences including RMLDVEKC, DTSSK, and QFEET motifs. The part of the RMLDVEKC motif and the entire DTSSK motif that have been identified by MotifFinder are RNA pol-like motifs. **b** Antigens are presented by APCs as antigenic peptides, which are recognized by the T-cell receptor (TCR; Signal 1). The second signal (Signal 2) is delivered when B7 (CD80 and CD86) on the APCs engage CD28 on the T cells

The structure of PD-L1 includes an extracellular domain followed by a transmembrane domain and an intracytoplasmic region [53]. As shown in Fig. 1b, the extracellular domain of PD-L1 consists of Ig variable distal and constant proximal regions. It is anchored to the membrane by a hydrophobic transmembrane sequence. The intracytoplasmic region consists of three conserved sequences including RMLDVEKC and DTSSK motifs, which are RNA pol-like motifs [57], and a QFEET motif. The DTSSK motif is a negative regulator of the RMLDVEKC motif, which is responsible for suppressing the phosphorylation of signal transducer and activator of transcription 3 in tumor cells [57].

The underlying mechanisms of the PD-1 signaling pathway are briefly summarized in Fig. 1a as PD-1 binds to PD-L1 suppressing ZAP70 and PI3K phosphorylation by recruiting Src homology 2 domain-containing protein tyrosine phosphatase (SHP)1 and SHP2 phosphatases to the ITSM and ITIM motifs in the intracellular tail [58]. Consequently, the TCR signaling cascade is terminated [59]. SHP1 can bind to the ITIM and ITSM motifs, whereas SHP2 preferentially binds to the ITSM [60, 61]. However, it is still unknown whether SHP1 and SHP2 compete to bind to the ITSM or both bind to the intracellular tail. The engagement of PD-L1 with PD-1 leads to phosphorylation of ITSM and SHP-2 recruitment. As a result, the phosphatidylinositol 3-kinase (PI3K)/Akt signaling pathway is suppressed [62, 63]. PI3K/Akt signaling pathway blockage further regulates a series of downstream cellular events including the activation of the mechanistic targets of rapamycin (mTOR), the activation of Bcl-Xl, the production of interleukin (IL)-2, and the activation of nuclear factor-κB as well as inhibits protein synthesis and cell growth. In addition, PI3K/Akt signaling pathway blockage degrades transcription factor FoxO1, which binds to the promoter site of PD-1 and increases its expression [31, 62].

**Fig. 2:**
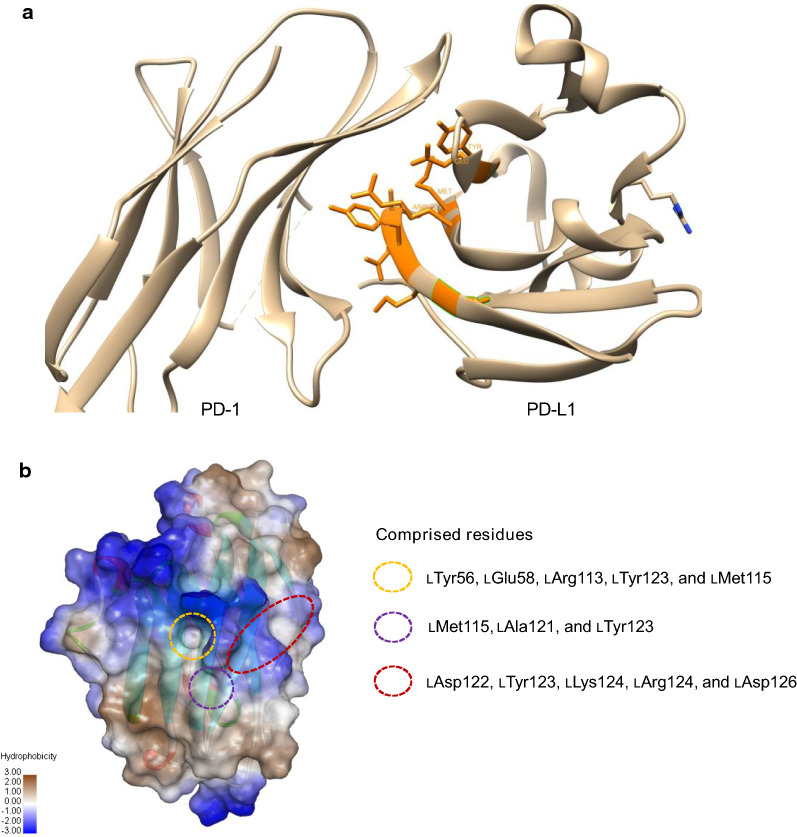
The structure of PD-L1 (4ZQK) and three main hot spots between PD-1 and PD-L1. **a** The structures of PD-L1 and PD-1. Amino acid residues in the main hot spots are labeled as orange color. **b** Three main hot spots are exhibited. The first hotspot includes lTyr56, lGlu58, lArg113, lMet115, and lTyr123. The second hotspot includes lMet115, lAla121, and lTyr123. The third hotspot is an extended groove formed by the main chain and the side chains spanning residues lAsp122 to lArg125, and is flanked by the side chain of lAsp26

The protein crystal structures of the PD-1/PD-L1 complex reveal that their interactions use large, hydrophobic surfaces of the extracellular domains [53]. Within the complex, PD-1 and PD-L1 are almost perpendicular to each other, facilitating interactions through the majority of the surface of their ‘‘front’’ strands. Currently, there are three identified hotspots on PD-L1 (Fig. 2). Two of three hotspots are regarded as drug binding pockets. The first hotspot is a classic pocket with a hydrophobic domain, which includes amino acid residues lTyr56, lGlu58, lArg113, lMet115, and lTyr123. This hotspot has a favorable size to accommodate an aromatic six-membered ring. The second hotspot with lMet115, lAla121, and lTyr123 residues can be effectively occupied by a branched aliphatic moiety, which can anchor with a terminal hydrogen bond donor moiety at the carbonyl oxygen of lAla121. The third hotspot is an extended groove formed by the main chain and the side chains spanning residues lAsp122 to lArg125, and is flanked by the side chain of lAsp26. This hotspot can provide multiple hydrogen bond donors/acceptors. However, it has a relatively shallow space, making it a difficult target for inhibitors of protein interactions.

Overall, it is challenging to target the interface of PD-1/PD-L1 because of its large and flat hydrophobic pockets (1970 A^2^) as compared to some other druggable proteins with deep hydrophobic pockets [53]. One of the rational designs for the discovery of PD-1/PD-L1 inhibitors is to evaluate the interactions between the leading compounds and these drug binding pockets using computational based screening methods, which can be further validated by in vitro and in vivo bioassays to eliminate false positive “hits”.

## Current methodologies for the development of PD-1/PD-L1 inhibitors

**Fig. 3:**
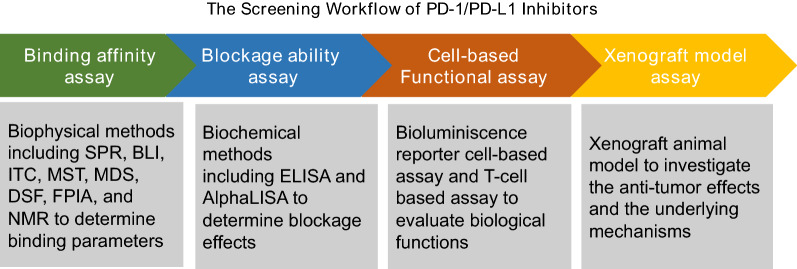
The screening workflow of PD-1/PD-L1 inhibitors. The identification of PD-1/PD-L1 inhibitors is required by using a series of assays including binding affinity assay, blockage ability assay, cell-based functional assay and xenograft model assay

Over the past decades, considerable research efforts have been dedicated to the development of small molecule inhibitors against PD-1/PD-L1 immune checkpoints [64–66]. Biophysical and biochemical assays along with cell-based assays have been developed to identify and evaluate the binding affinity between these inhibitors and PD-1/PD-L1, and their blockage effects toward PD-1/PD-L1 interactions. A workflow for screening potential inhibitors of PD-1/PD-L1 is shown in Fig. 3.

Binding affinity is one of the most critical parameters to measure the capacity of potential inhibitors binding to PD-1/PD-L1 proteins. PD-1/PD-L1 interactions can be characterized by a series of methods summarized in this review. These biophysical methods are usually performed at the protein level. Although these methods may lead to indefinite parameters regarding the dissociation constant (K_D_) [53, 55, 67, 68], binding affinity measurement is still usually required to identify small molecule inhibitors against PD-1/PD-L1.

### Surface plasmon resonance (SPR)

SPR is an optical biosensor technology based on the evanescent wave phenomenon to measure changes in the refractive index of biosensor [69]. The light generated by the light source hits the biosensor and prism. Analyte flows through the channel and binds to the target protein, leading to a shift in the refractive index of the biosensor. The interactions between analyte and proteins are monitored in a real-time manner and the amount of bound proteins and rates of association and dissociation are measured with high precision. SPR is widely used for determining intermolecular interactions. PD-1/PD-L1 interactions are based on their extracellular domains and their interactions include hydrophobic and polar effects. Small molecule inhibitors with blockage effects against PD-1/PD-L1 interactions bind to their extracellular interface. Therefore, SPR is an ideal tool to measure the binding affinity between inhibitors and PD-1/PD-L1. SPR can also determine the real-time kinetic constants between inhibitors and PD-1/PD-L1, which requires the immobilization of PD-1/PD-L1 protein on certain biosensors. His-tagged and tag-free PD-1/PD-L1 have been widely immobilized on the biosensors using amine coupling methods [44, 65]. For instance, Yang and colleagues developed an SPR technology-based screening method that has successfully screened caffeoylquinic acids as PD-1/PD-L1 inhibitors by immobilizing tag-free PD-1/PD-L1 extracellular fragment on the CM5 biosensor chip [65]. An advantage of SPR is that no modification is required for the target proteins as compared to other screening methods including NMR-based AIDA and HTRF.

### Biolayer interferometry (BLI)

BLI, similar to SPR, is a label-free technique monitoring real-time biomolecule interactions [70]. The working mechanism of BLI is similar to SPR as it detects the changes of the optical interference patterns on the protein-coated biosensors that are generated by mass changes from the interactions between analyte and protein [70]. Less protein is required for BLI measurement, which facilitates high-throughput screening with great potential to screen small molecule inhibitors against PD-1/PD-L1. Unlike SPR which detects biomolecular interactions under flow conditions, BLI is conducted under non-flow conditions which impair its ability to depict the kinetic profiles.

### Isothermal titration calorimetry (ITC)

ITC is a useful method to characterize the thermodynamic parameters of interactions between analytes and proteins [71]. The binding events are accompanied by changes of enthalpy (∆*H*). Analyte-protein interactions driving the process and parameters including stoichiometry of binding (n), the binding constant (Ka), K_D_, ∆*H*, and entropy (ΔS) can be determined. PD-1/PD-L1 interactions exhibit a favorable Δ*H*obs and TΔS and their binding is driven entropically [53]. However, Pascolutti and colleagues reported that the driving force of wild-type PD-1/PD-L1 exhibits an entropic component [72]. An advantage for ITC measurement is that it does not require immobilization, protein modification, or fluorescent labeling. It is also an approach that can measure all binding parameters in a single assay. However, ITC is not suitable for high throughput screening due to being time-consuming with high sample consumption.

### Microscale thermophoresis (MST)

MST is a suitable technology for determining the intramolecular interactions with less sample consumption [73]. MST is based on the directed movement of molecules along a microscopic temperature gradient [74]. Changes in their hydration shell, charge, or size can be determined in this process. MST technology requires two binding partners, one is labeled with fluorescence dye and the other one is free-labeled [74]. MST does not require immobilization. Intermolecular interactions can be measured under physicochemical conditions or biological solutions. In addition, protein purification is also not required to access the protein of interest [75]. However, the binding partner labeled with hydrophobic fluorescence may lead to non-specific binding. Consequently, the bias of the results might be observed due to the indiscriminate fluorescent labeling.

MST is applied to determine PD-1/PD-L1 binding affinities [67] whereby cell lysate is extracted from CHO-K1 cells that express PD-1-eGFP or PD-L1-eGFP, to prepare the fluorescently labeled binding partner. PD-L1 or PD-1 protein is used as label-free binding partners. The K_D_ value of 7.2 μM ± 1.9 μM between hPD-1 and hPD-L1-eGFP is obtained using MST [67], which is similar to SPR assay (K_D_ value of hPD-1/hPD-L1 = 8.2 ± 0.1 μM) [53, 76]. Therefore, MST technology highlights its potential application for studying the interactions between PD-1/PD-L1 and their inhibitors.

### Differential scanning fluorometry (DSF)

DSF is an excellent screening assay to discover low-molecular-weight ligands with binding affinities for target proteins by monitoring the amount of the fluorescent dye that binds to the protein [77]. Ligand is added into the solution containing protein and fluorescent dye in the polymerase chain reaction (PCR) microplates. Fluorescent intensities are measured as the temperature is gradually raised by the PCR instrument [78]. The binding of PD-1/PD-L1 inhibitors induces thermal stabilization of PD-1/PD-L1, which is proportional to the inhibitors’ affinity [79]. DSF is suitable for high-throughput screening due to the small amount and low concentrations needed for protein binding. However, impurities (e.g. detergent molecules) have to be excluded from the reaction system. In addition, the interactions between fluorescent dye and target proteins may interfere with the detection results. Recently, it was reported that proteins that have already been labeled with green fluorescent can be applied to avoid the interactions with the fluorescent dye [80].

### Fluorescence polarization immunoassay (FPIA)

FPIA is based on the principle of fluorescence anisotropy. As a homogenous assay, it determines the rotational and translational motion of excited fluorescent molecules in the reaction mixture [81]. It is a rapid and quantitative method to detect several biomolecular interactions and enzyme activities. This assay is a feasible mix-and-read method with fewer reagents required, which is suitable for high-throughput screening of peptides or nucleotide sequences binding to PD-1/PD-L1. For instance, it has been successfully demonstrated that FPIA can be applied to analyze the affinity between self-inhibitory peptides (refers to peptides disrupting the PD-1/PD-L1 complex formation) and PD-1 [82]. A major disadvantage of FPIA is that the protein–protein interactions containing extensive interfaces can lead to low sensitivity for detecting biomolecules that are disproportionately important for the affinity of the interactions. In this case, competitive binding assays with specific fluorescence polarization probes can be applied to study the interactions between the molecules (e.g. PD-1/PD-L1 inhibitor) and their featured interfaces [83, 84].

### Nuclear magnetic resonance (NMR)

NMR is a powerful tool to determine the structure, dynamics, and biomacromolecule interactions. NMR can also detect the binding affinities of protein targets with small molecules that have a broad affinity range [85, 86]. It can detect weak intermolecular interactions, which makes it a valid screening tool for low-affinity fragments [86]. However, binary screening NMR does not give information on whether the small molecules can exert blockage effects on protein–protein interactions. To overcome this limitation, Musielak and colleagues described an NMR competitive assay, termed as weak-antagonist induced dissociation assay-NMR (w-AIDA-NMR). In this competitive assay, lead compounds with capacity of dissociating protein–protein interactions are depicted by the strength of their binding affinities with protein components involved in the protein–protein interactions [85, 87]. The K_D_ value of PD-1/PD-L1 complex is approximately 8 μM, which might be too strong for the NMR-based screening for “weak” fragments, as these fragments exhibit lower affinities with 2 to 3 orders of magnitude. Therefore, instead of using PD-1, PD-1 mutant can be applied to estimate the K_D_ value of fragments with PD-1/PD-L1. The K_D_ values between fragments and PD-L1 by using w-AIDA-NMR method are similar to the corresponding data from the HTRF assays, supporting the reliability of the w-AIDA-NMR method. In addition, some small molecule PD-L1 inhibitors that block the PD-1/PD-L1 interactions have also been characterized using AIDA NMR [64, 88, 89]. Interestingly, a combination of AIDA-NMR, PD-1/PD-L1 structure-based design, and fragment merging approaches creates novel chemotypes as a starting point for the development of small molecule inhibitors against PD-1/PD-L1 [88]. Recently, high-field NMR spectrometers have been developed to improve the NMR's sensitivity and resolution [90], which highlights the potential application of NMR-based methods in large-scale screening.

### The enzyme-linked immunosorbent assay (ELISA) and alphaLISA

ELISA is a solid-phase type of enzyme immunoassay to detect the presence of proteins using antibodies against the proteins to be measured [91]. Because PD-L1 has a strong binding affinity with PD-1, PD-1/PD-L1 pair ELISA can be applied for screening small molecules with blockage effects towards PD-1/PD-L1 interactions. Briefly, PD-1 or PD-L1 protein (or PD-1/PD-L1 extracellular domain) is coated by incubation with biotin labelled-PD-L1 or PD-1 with or without the small molecules of interest. Next, streptavidin–horseradish peroxidase and colorimetric horseradish peroxidase substrates are added. The inhibitory abilities of small molecules towards PD-1/PD-L1 interactions are determined by comparing the optical density values among the experimental groups. Although ELISA is a widely used detection platform for PD-1/PD-L1 inhibitors, it requires multiple procedure steps (e.g. washes) with a relatively narrow dynamic range (typically 2 logs). Therefore, more than one sample dilution is required, which makes PD-1/PD-L1 pair ELISA less feasible to adapt for high-throughput screening.

Alternatively, AlphaLISA is a homogeneous immunoassay that can be used to screen for PD-1/PD-L1 inhibitors in a high-throughput manner [92]. AlphaLISA is a bead-based immunoassay without the requirement of ‘wash’. Therefore, it avoids washing times thereby reducing the total assay time as compared to ELISA. The principle of the AlphaLISA method is based on luminescent oxygen-channeling chemistry. AlphaLISA consists of donor beads and acceptor beads. Streptavidin-coated donor beads are used to bind biotinylated-PD-L1, and anti-His acceptor beads are used to bind to His-tagged PD-1. Donor beads and acceptor beads interact with each other due to the strong binding affinity between PD-1 and PD-L1. Donor beads contain a photosensitizing agent that can be illuminated by a wavelength of 680 nm generating singlet oxygen, which initiates a cascade reaction with the acceptor beads. Consequently, the acceptor beads will generate a remarkable signal amplification (at 615 nm) by singlet oxygen released from the donor beads. Small sample volumes (1–5 μL) with high sensitivity and wide dynamic ranges (typically 3 logs) are required in the AlphaLISA assay. Therefore, it is an ideal platform for high-throughput screening.

### Bioluminescent reporter cell-based assay

Bioluminescent reporter cell-based assay, which consists of two engineered cell lines including PD-1 effector cell line and PD-L1 aAPC/CHO-K1 cell line, can be used for screening the PD-1/PD-L1 inhibitors [93]. PD-1 effector cell line is constructed on Jurkat T cell line that stably expresses PD-1 by transfection of luciferase reporter plasmids containing NFAT response element. PD-L1 aAPC/CHO-K1 cell line is constructed on CHO-K1 cell line that expresses PD-L1 by engineering cell surface proteins to activate cognate TCRs without antigen. In the co-culture system, PD-1 binds to PD-L1 and subsequently suppresses the TCR signaling and luminescence mediated by NFAT response element. The presence of PD-1/PD-L1 inhibitors blocks the PD-1/PD-L1 interactions, leading to the reactivation of TCR signaling and luminescence. Quantification of TCR activation with or without PD-1/PD-L1 inhibitors is measured by the intensity of luciferase activity. In addition, the NF-κB reporter assay is an alternative option for the NFAT response element reporter system. However, it is preferable to use the NFAT response element reporter system because the NF-κB signaling is a less specific marker of the CD3 dependent T cell activation that can be activated by other stimuli7 [94, 95].

Unlike the other aforementioned biophysical and biochemical assays that cannot evaluate the functional impact of small molecules on PD-1/PD-L1 interactions, bioluminescent reporter cell-based assay has an advantage of assessing the biological functions of PD-1/PD-L1 inhibitors by measuring the activation of NFAT signaling pathway. In addition, this commercialized assay is a labor- and time-efficient tool, which is suitable for high-throughput screening. Moreover, bioluminescent reporter cell-based assay has less variation as compared to primary cell-based assays [96]. However, the current bioluminescent reporter cell-based assays cannot provide information of antigen-specific or multiparametric interactions. Due to PD-1/PD-L1 mediated downstream signaling transductions involved in many proteins [31], the bioluminescent reporter cell-based assay is insufficient to evaluate the functions of PD-1/PD-L1 inhibitors on the signaling transduction-related proteins.

### T cell-based assay

Although a cell-free assay system can be used to evaluate the basic biological functions of PD-1/PD-L1 inhibitors, further biological effects of leading inhibitors on PD-1/PD-L1’s physiological properties, including their subcellular localization, or functional changes upon stimulation, might not be evaluated sufficiently with cell-free assays alone. To evaluate the bioactivities and complicated physiological functions of PD-1/PD-L1 inhibitors, T cell-based assays are often used.

T cell-based assays consist of effector cells expressing PD-1, cells presenting PD-L1, and the activation signal (CD3 activator) for effector cells. Several methods for the development of inhibitors targeting PD-1/PD-L1 using T cell-based assays have been reported [47]. The activation of CD3 (Signal 1) is an essential step for the activation of PD-1 effector cells in this assay. The TCR/CD3 can be expressed by the effector cells and activated by several biological components including peptide/MHC complex on the target cells, superantigen in the presence of APCs expressing MHC II, soluble CD3ε antibodies, and activator cells expressing transmembrane aCD3ε. In the T cell-based assays, tumor cells or target cells expressing tumor-associated antigen are often used [97]. In these assays, the presence of effector cells express tumor-associated antigen-specific CAR containing the CD3ζ signaling domain, or TCR/CD3 effector cells with CD3 antibodies and tumor-associated antigen antibodies, leads to the dependent activation of CD3 in the effector cells.

In the T cell-based assays, immobilized cell lines are preferable to avoid issues with accuracy and reproducibility associated with primary cells [98, 99]. For instance, the immobilized Jurkat human T cell line, a commonly used T cell line, has been successfully developed to measure the CD3 dependent T cell activation [100]. In addition, the Jurkat human T cell line is suitable for genetic engineering, which can be applied for evaluating the biological effects of small molecules targeting PD-1. Versteven and colleagues developed an antigen-specific and high-throughput T cell-based assay by using a genetically modified TCR-deficient Jurkat T cell line that is also transduced with PD-1 plasmid [101].

T cell-based assays are widely used to evaluate the blocking abilities and biological functions of PD-1/PD-L1 inhibitors [102]. Although the binding abilities of small molecule inhibitors against PD-1/PD-L1 are usually analyzed by biophysical and biochemical assays, T cell-based assays are also used to evaluate their blocking abilities based on flow cytometry method [103]. To evaluate the blocking abilities of PD-1/PD-L1 small molecule inhibitors, cell co-culture based assays or single-type cell incubated with PD-1 or PD-L1 proteins are often used. For instance, small molecule inhibitors can be incubated in a co-culture system with T cells expressing PD-1 and APCs/tumor cells expressing PD-L1. The blocking abilities of small molecule inhibitors can be evaluated by measuring PD-1/PD-L1 expression using flow cytometry [104]. Similarly, in a single-type cell incubated with PD-1 or PD-L1 protein, the blocking affinity of small molecule inhibitors against PD-1 or PD-L1 protein is measured by the qualification of fluorescence intensity.

The primary aim for using T cell-based assay is to verify the biological functions of PD-1/PD-L1 inhibitors. In the tumor microenvironment, overexpression of PD-1 leads to T cell dysfunction, whereas PD-1/PD-L1blockage reactivates T cell's biological functions [105]. The functional assays need a co-culture system consisting of PD-1 expressing cells and PD-L1 expressing cells. It is based on the change of T cell dysfunction in the presence of small molecules targeting PD-1/PD-L1. The functional assays of T cells include measurements of cell proliferation, T cell-related cytokine release (IL-2 and interferon (IFN)-γ), and the change of PD-1 downstream events including signaling proteins and their phosphorylation [106]. For instance, low proliferative capacity is a key character of T cell dysfunction [54], and cell proliferation is one of the most used assays to evaluate the biological functions of PD-1/PD-L1 inhibitors. In addition, the detection of IL-2 and IFN-γ are also widely used in the functional assay as IL-2 and IFN-γ are essential for T cell proliferation and activity, respectively [107, 108]. Furthermore, signaling proteins involved in the PD-1/PD-L1 axis-mediated signaling transductions can be investigated to evaluate the biological effects of PD-1/PD-L1 inhibitors.

## Natural product-derived PD-1/PD-L1 inhibitors

Most mAbs have inherent shortcomings including limited permeability, irAEs, immunogenicity, and high cost, as compared to small molecules derived PD-1/PD-L1 inhibitors [109, 110]. Small molecule inhibitors usually have less side effects, shorter biological half-life, and are less expensive with easier administration routes. Several published review articles have summarized the advantages of various synthetic small molecule PD-1/PD-L1 inhibitors [64, 66, 111]. Recently, several natural product-derived small molecules with blockage effects against PD-1/PD-L1 interactions have been reported. Instead of elaborating on all current small molecule inhibitors, herein we summarize natural product-derived PD-1/PD-L1 inhibitors with an emphasis on the screening methodologies that were applied for their identification.

### Macrocyclic compounds

#### Gramicidin derivatives from *Bacillus brevis*

**Fig. 4:**
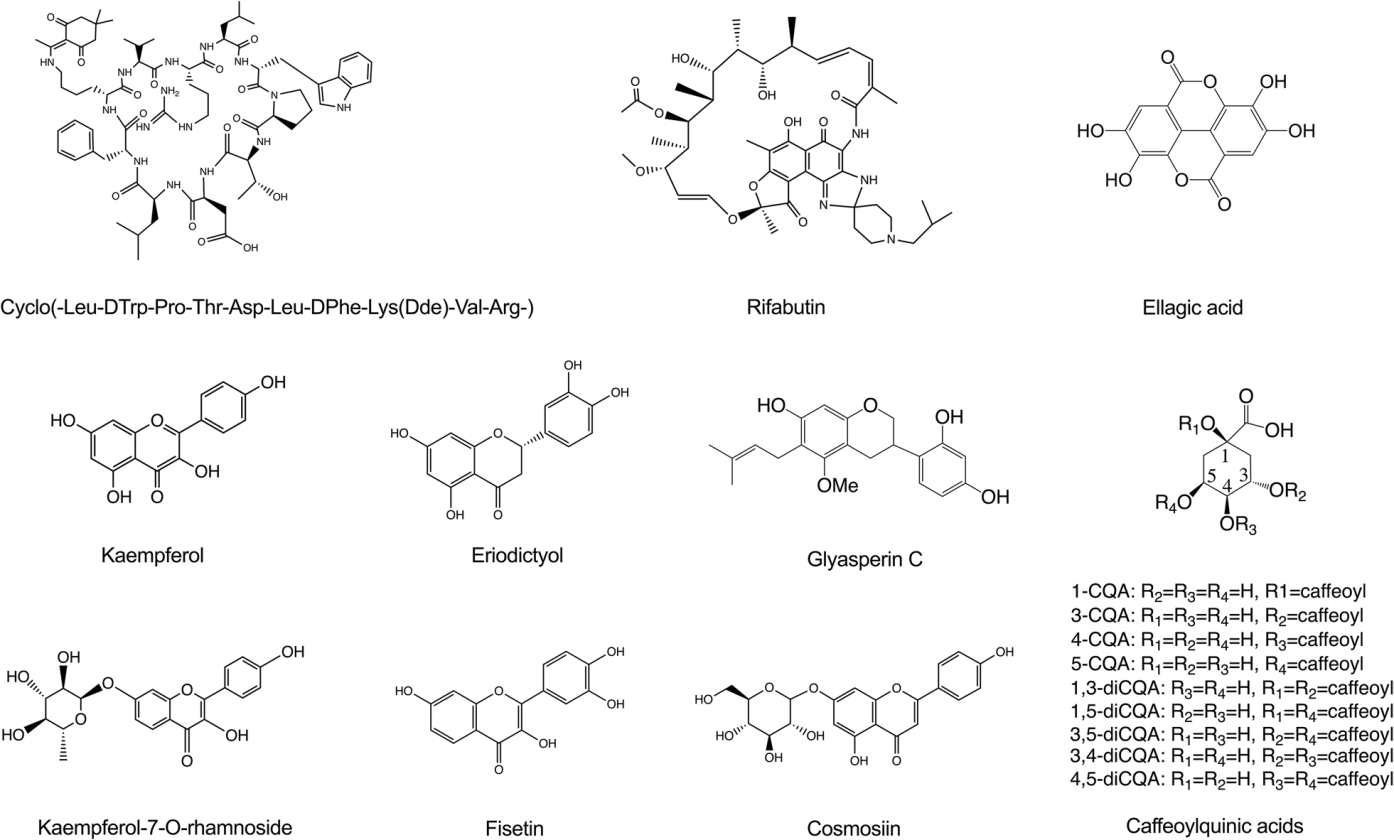
Chemical structures of natural products based PD-1/PD-L1 inhibitors including cyclo(-Leu-DTrp-Pro-Thr-Asp-Leu-DPhe-Lys(Dde)-Val-Arg-), rifabutin, kaempferol, kaempferol-7-*O*-rhamnoside, eriodictyol, fisetin, glyasperin C, cosmosiin, ellagic acid, and caffeoylquinic acids

Gramicidin S is a natural decacyclopeptide consisting of two repeating pentapeptides as cyclo(-Val-Orn-Leu-D-Phe-Pro-)2, which imposes a unique amphiphilic structure with hydrophilic and hydrophobic residues on the opposing side of cyclopeptide plane ring. Sun and co-workers hypothesized that gramicidin S’s amphiphilic structure can be complementary to the interface of PD-L1/PD-L1 thereby facilitating their binding capacity [112]. An in vitro binding assay (HTRF) was determined to evaluate the blockage efficacy of cyclopeptides towards PD-L/PD-L1 binding and gramicidin S exhibited a weak blockage efficacy of 6.86%. They further chemically synthesized a series of cyclopeptides using the skeleton of gramicidin S [112]. Among the synthesized gramicidin S derivatives, Cyclo(-Leu-DTrp-Pro-Thr-Asp-Leu-DPheLys(Dde)-Val-Arg (Fig. 4) exhibits the most potent blockage efficacy of 95.8% at 20 µM against PD-1/PD-L1 interactions. It had the lowest IC_50_ value of 1.42 µM against PD-1/PD-L1 interactions based on the co-immunoprecipitation assay. Co-administration of Cyclo(-Leu-DTrp-Pro-Thr-Asp-Leu-DPheLys(Dde)-Val-Arg (40 mg/kg) by intraperitoneally injection (ip) with anti-CD8 antibody suppressed the tumor volume (54.8%) and tumor weight (64.9%) in a B16F10 tumor bearing animal model. Immunohistochemistry staining showed that treatment with Cyclo(-Leu-DTrp-Pro-Thr-Asp-Leu-DPheLys(Dde)-Val-Arg enhanced the percentage of CD3+ T cells and CD8+ T cells in the tumor tissues. In addition, the binding properties of the most promising cyclopeptide were well characterized using a panel of biochemical, biophysical, and cell-based assays including SPR, Western blotting (WB), NMR, circular dichroism (CD), co-immunoprecipitation, and molecular docking. The key findings in this study are summarized in Table 1.

**Table 1:** A summarize of natural product-derived PD-1/PD-L1 inhibitors

Natural products			Methodology	Key finding(s)
Name	Type	Sub-type		
Amphotericin B	Macrocyclic	Macrolide	AlphaLISA; MD	Not active
Bacitracin		Cyclic peptide		
Everolimus		Macrolide		
Clarithromycin		Macrolide		
Cyclosporin A		Cyclic peptide		
Actinomycin D		Cyclic peptide		Weak PD1/PD-L1 inhibitor (less than 20% inhibition at 50 µM)
Cynocobalamin		Porphyrin		
Bryostatin		Macrolide		
Candicidin		Macrolide		
Geldanamycin		Polyketide		
Ivermectin B1a		Macrolide		
Macbecin		Ansamycin		
Metocurine		Alkaloid		
Monocrotaline		Alkaloid		
Nystatin		Macrolide		
Plerixafor		Bicyclam		
Sirolimus		Macrolide		
Troleandomycin		Macrolide		
Rifampin		Ansamycin		PD1/PD-L1 inhibition was 47.9% at 50 µM
Rifabutin				PD1/PD-L1 inhibition was 66.7% at 50 µM IC_50_ was 25 µM
Rifapentine				PD1/PD-L1 inhibition was 52.1% at 50 µM
Rifamycin SV				PD1/PD-L1 inhibition was 34.5% at 50 µM
Formyl rifamycin				PD1/PD-L1 inhibition was 40.2% at 50 µM
Rifaximin				PD1/PD-L1 inhibition was 24.0% at 50 µM
Gramicidin S	Macrocyclic	Cyclic peptide	HTRF; NMR; SPR; CD; MD	PD1/PD-L1 inhibition was 6.86% at 20 µM
Gramicidin S derivative				PD1/PD-L1 inhibition was 95.8% at 20 µM; IC_50_ was 1.42 µM Conserved the β-sheet conformation of the gramicidin S skeleton *K*_D_ was 1.66 mM and 5.67 µM for PD-1 and PD-L1, respectively
Kaempferol	Phenolic	Flavonoid	ELISA; BLI; SPR Cell based assay MD	IC_50_ for blocking PD-1/PD-L1 was 7.797 µM Cellular PD-1/PD-L1inhibition IC_50_ was 14.46 µM Calculated binding energy was -5.4 and -5.0 kcal/mol for PD-1 and PD-L1, respectively
Kaempferol-7-*O*-rhamnoside		Flavonoid		Cellular PD-1/PD-L1inhibition IC_50_ was 14.46 µM *K*_D_ was 31.1 and 19.7 µM for PD-1 and PD-L1, respectively Calculated binding energy was -5.6 and -5.3 kcal/mol for PD-1 and PD-L1, respectively
Cosmosiin	Phenolic	Flavonoid	ELISA; BLI Cell based assay MD	Increased T-cell functional activity by 1.91-fold; Had *K*_D_ value of 386 and 85 µM for PD-1 and PD-L1, respectively Fit to a 1:1 binding model to PD-1 and PD-L1; Had a predicted binding affinity of − 6.2 and − 5.8 kcal/mol for PD-1 and PD-L1, respectively
Apigenin		Flavonoid		Increased T-cell functional activity by 2.03-fold
Eriodictyol	Phenolic	Flavanone	ELISA	Had an IC_50_ of 0.04 µM for PD-1/PD-L1
Fisetin		Flavonol		Had an IC_50_ of 0.04 µM for PD-1/PD-L1
Glyasperin C	Phenolic	Isoflavan	HTRF	Had an PD-1/PD-L1 inhibition rate of 64.3% at 100 µM
Caffeoylquinic acid	Phenolic	–	SPR	*K*_D_ = 1.24 × 10^−5^ M for PD-1; not detected for PD-L1
3-*O*-caffeoylquinic acid		Caffeoylquinic acid		*K*_D =_ 1.95 × 10^−6^ M for PD-1; 1.71 × 10^−5^ M for PD-L1
4-*O*-caffeoylquinic acid		Caffeoylquinic acid		*K*_D_ = 5.07 × 10^−6^ M for PD-1; not detected for PD-L1
5-*O*-caffeoylquinic acid		Caffeoylquinic acid		*K*_D_ = 1.68 × 10^−5^ M for PD-1; 8.13 × 10^−5^ M for PD-L1
Ellagic acid	Phenolic	–	ELISA WB Cell based assay	Blocked PD-1/PD-L1 binding with an IC_50_ value of 22.92 μg/mL Bound to PD-1 and PD-L1 in WB;
ZINC 67,902,090	Heterocyclic	Pyrrolidine-oxadiazole	AlphaLISA WB MD	PD-1/PD-L1 inhibition potency was 30% as compared to BMS-202
ZINC 12,529,904				PD-1/PD-L1 inhibition potency was 40% as compared to BMS-202

#### Ansamycin antibiotic

Patil et al. used an AlphaLISA assay to screen the inhibitory effects of FDA-approved macrocyclic drugs against PD-1/PD-L1 interactions [113]. A collection of 20 macrocyclic compounds including actinomycin D, amphotericin B, bacitracin, bryostatin, candicidin, clarithromycin, cyclosporin A, cyanocobalamin, erythromycin, everolimus, geldanamycin, ivermectin B1a, macbecin, metocurine, monocrotaline, nystatin, plerixafor, rifampin, sirolimus, and troleandomycin was screened at a concentration of 50 µM using the AlphaLISA assay. Among these macrocyclics, only rifampin (Fig. 4), an ansamycin type of antibiotic, effectively blocked the interactions between PD-1 and PD-L1 (blockage efficacy = 47.9%) whereas the other compounds were less effective (blockage efficacy < 20%). Four additional rifampin analogs including rifabutin, rifapentine, rifamycin SV, and 3-formyl rifamycin were selected for further evaluation. Rifampin analogs (50 µM) showed promising blockage efficacy ranging from 24 to 66.7%, in which rifabutin was the most active macrocyclic antibiotic with an IC_50_ value of 25 µM (Table 1). In addition, molecular docking demonstrated that rifabutin is able to form a stable ligand–protein complex facilitated by several molecular forces including π–π stacking interaction and hydrogen bonding. However, binding affinities between these ansamycin antibiotics and PD-1/PD-L1 proteins are not reported.

### Phenolic compounds

#### Kaempferol and kaempferol-7-*O*-rhamnoside

Kaempferol and its glycosides including kaempferol-3,7-dirhamnoside and kaempferol-7-*O*-rhamnoside, are flavonoids from *Geranium thunbergia* (*Geranii Herba* extract) with reported antitumor activities [114]. In vitro assays were used to demonstrate that kaempferol and kaempferol-7-*O*-rhamnoside are able to block PD-1/PD-L1 interactions. Competitive ELISA assays were used to measure the inhibitory effects of kaempferol and kaempferol-7-*O*-rhamnoside (Fig. 4) on the PD-1/PD-L1 interactions, which were supported by cell co-culture (Jurkat T/CHO-K1 cells) assay. The EC_50_ values of kaempferol and kaempferol-7-*O*-rhamnoside were 16.46 and 15.37 μM, respectively, against PD-1/PD-L1 interactions in a dose-dependent manner. The direct binding between kaempferol and PD-1 or PD-L1 were measured by obtaining the binding kinetics including the K_D_, ka, and kd using BLI and SPR technologies. In addition, a computational-based approach was used to map the binding site of kaempferol and kaempferol-7-*O*-rhamnoside on PD-1 or PD-L1 and calculate the binding energy between the ligands and proteins.

#### Apigenin and cosmosiin from *Salvia plebeia*

Choi et al. reported that *Salvia plebeia* R. Br. extract (SPE) blocked the interactions between PD-1 and PD-L1 [115]. Two flavonoids including apigenin and cosmosiin (Fig. 4) from SPE showed blockage effects against the interactions between PD-1 and PD-L1 in a cell-based assay (aAPC/CHO-K1 cells) and a competitive ELISA assay. PD-L1 aAPC/CHO-K1 cell co-culture based assay demonstrated that EC_50_ values of SPE and SPE-ethyl acetate fraction were 27.2 mg/mL and 1.08 mg/mL, respectively, against PD-1/PD-L1 interactions. In addition, cosmosiin, identified as the strongest PD-1/PD-L1 inhibitor among 7 SPE fractions, was able to directly bind to PD-1 and PD-L1 with a K_D_ value of 386 and 85 µM, respectively, in the BLI assay. Computational docking was then determined to predict cosmosiin’s binding capacity to PD-1 and PD-L1, showing a binding energy of -6.2 and -5.8 kcal/mol, respectively (Table 1). Moreover, the inhibitory effect of SPE on PD-1 and PD-L1 was further supported by in vivo assays using a humanized PD-L1 knock-in MC38 tumor-bearing animal model. Treatment of SPE at doses of 100 and 300 mg/kg exhibited tumor inhibition rates of 44.9 and 77.8%, respectively, in a dose-dependent manner on day 16. In addition, treatment of SPE (300 mg/kg) enhanced the infiltration of CD8+ T cells in the tumor tissues.

#### Eriodictyol and fisetin from *Rhus verniciflua* Stokes extract

Li and colleagues screened 800 herbal extracts for the PD-1/PD-L1 inhibition capacity, which led to the identification of *Rhus verniciflua* Stokes extract as an active inhibitor using competitive ELISA [116]. Four phenolic compounds including eriodictyol, fisetin, quercetin, and liquiritigenin were isolated from the *Rhus verniciflua* Stokes extract with PD-1/PD-L1 blocking effect. Eriodictyol and fisetin showed the most potent inhibitory effect in the competitive ELISA with an IC_50_ value of 0.04 and 0.4 µM, respectively. However, the binding affinity between eriodictyol or fisetin and PD-1/PD-L1 was not reported.

#### Glyasperin C from *Glycyrrhiza uralensis*

Bao et al. reported the isolation of a flavonoid, glyasperin C (Fig. 4), from *Glycyrrhiza uralensis* and its PD-1/PD-L1 inhibitory effect using a commercially available homogeneous time resolved fluorescence (HTRF) assay [117]. The isolated compounds showed PD-1/PD-L1 inhibition ratios ranging from 30 to 65% at 100 µM.

#### Ellagic acid from black raspberry (*Rubus coreanus* Miquel) extract

Kim et al. reported that a black raspberry (*Rubus coreanus* Miquel) extract (RCE) interrupted the binding of PD-1 and PD-L1 with an IC_50_ value of 83.8 ± 4.7 μg/mL in the competitive ELISA assay [118]. PD-L1 aAPC/CHO-K1 cell co-culture based assay revealed that RCE increased the production of IL-2 by 1.8-fold with an EC_50_ value of 56.15 ± 14.35 μg/mL, as compared to the control group. The inhibitory effect of RCE on PD-1/PD-L1 interaction was further supported by in vivo data using a humanized PD-L1 knock-in MC38 tumor-bearing animal model, in which oral administration of RCE (50 and 100 mg/kg/day) exhibited tumor inhibition rates of 66.94% and 73.81%, respectively, on day 21. In addition, the major phytochemical in RCE was identified as ellagic acid (Fig. 4) and its effects on PD-1 and PD-L2 interaction were evaluated using in vitro assays including competitive ELISA, WB pull-down, and cell-based assays (PD-1 Jurkat effector cell/ PD-L1 CHO-K1 cell). Ellagic acid was shown to block PD-1/PD-L1 interaction in a concentration-dependent manner with an IC_50_ value of 22.92 μg/mL (Table 1). In addition, ellagic acid-conjugated sepharose 4B beads pull-down assay showed that ellagic acid was able to directly bind PD-1 and PD-L1 and interrupt their binding capacity [118].

#### Caffeoylquinic acid derivatives

Caffeoylquinic acid and its derivatives (Fig. 4) with a caffeoyl group attached to the − 3, − 4, and − 5 position of quinic acid, respectively, were identified as PD-1/PD-L1 inhibitors using SPR spectroscopic method [65]. The K_D_ values of caffeoylquinic acid and its derivatives on PD-1 and PD-L1, ranged from 0.507 × 10^–5^ to 1.68 × 10^–5^ M and from 1.71 × 10^–5^ to 8.13 × 10^–5^ M, respectively, as determined by SPR (Table 1). In addition, a competitive SPR assay was used to compare the binding capacity between quinic acid derivatives with one or two caffeoyl group(s) and PD-1. It was concluded that, as compared to dicaffeoylquinic acids, mono-caffeoylquinic acid derivatives had a stronger binding affinity with PD-1 and PD-L1.

### Heterocyclic compounds

Several heterocyclic compounds containing nitrogen atoms have been reported to show blockage effects against PD-1/PD-L1 interactions. Using in silico virtual screening methods, Lung et al. reported that two pyrrolidine-oxadiazole derivatives including (3S,3aR,6S,6aR)-N6-[4-(3-fluorophenyl)-pyrimidin-2-yl]-N3-(2-pyridylmethyl)-2,3,3a,5,6,6a-hexahydrofu (ZINC ID#67902090) and 1-isopropyl-3-[(3S,5S)-1-methyl-5-[3-(2-naphthyl)-1,2,4-oxadiazol-5-yl]pyrrolidin-3-yl]urea (ZINC ID#12529904) were identified as PD-1/PD-L1 inhibitors among 180,000 natural compounds from the ZINC12 database [119]. The inhibitory effects of ZINC 67,902,090 and 12,529,904 were evaluated by the AlphaLISA binding and PD-L1 dimer formation assays. AlphaLISA binding assays demonstrated that ZINC 67,902,090 and 12,529,904 have the potencies of 30 to 40% for inhibiting the PD-1/PD-L1 interaction, as compared to BMS-202 (100%). PD-L1 dimer formation assay showed that ZINC12529904 significantly promoted the amount of PD-L1 dimer, whilst ZINC 67,902,090 only slightly increased the amount of PD-L1 dimer. The binding mode of these two compounds was supported by the molecular docking study but their direct binding affinities were not investigated.

## Perspective

In 2018, the Nobel Prize in Physiology or Medicine was awarded to James Allison and Tasuku Honjo for their discovery of immune checkpoint therapy [120, 121]. PD-1 functions as a T-cell brake and the activation of PD-1/PD-L1 suppresses T cell’s proliferation, survival, and activity in the tumor microenvironment [31]. Clinical studies supported that PD-1/PD-L1 blockage can effectively introduce durable antitumor immune responses with less toxicity in many types of cancers [16]. Currently, the majority of approved PD-1/PD-L1 inhibitors are mAbs [16, 109] while the development of small molecule inhibitors directly blocking PD-1/PD-L1 interactions is still in the stage of infancy.

**Fig. 5:**
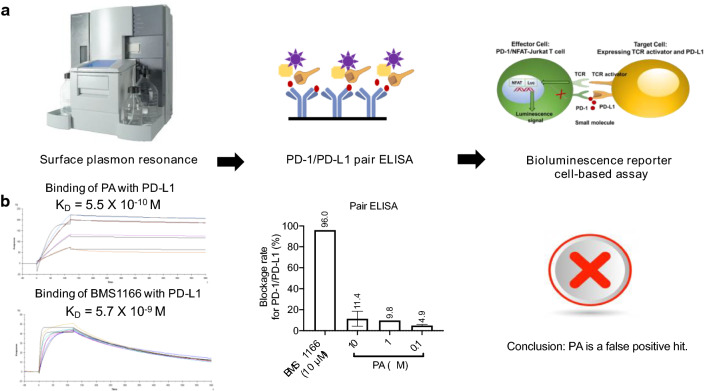
A workflow was established for screening PD-1/PD-L1 inhibitors. **a** SPR technology was performed to evaluate binding affinities followed by PD-1/PD-L1 pair ELISA assay. Once the inhibitors exert blockage effects on PD-1/PD-L1 interactions, bioluminescence reporter cell-based assay will be applied for determining their biological functions. **b** The binding and inhibitory effects of Punicalagin (PA) and BMS1166 against human PD-L1 protein assessed using SPR and PD-1/PD-L1 pair ELISA assays, respectively. PA or BMS1166 was allowed to flow over Fc-PD-L1 captured on a flow cell as well as on a reference cell of Series S Sensor Chip

Over the past decade, with a more advanced understanding of PD-1/PD-L1 interactions and the underlying mechanisms, there has been an explosion of interest in the development of bioassays that can be applied for screening small molecule inhibitors against PD-1/PD-L1 [64, 88, 101, 102, 104]. Biophysical and biochemical assays are powerful for screening the promising "hits" and for characterizing the binding parameters between identified "hits" and PD-1/PD-L1. Assays including ELISA, alphaLISA, bioluminescent reporter cell-based assays, and T-cell based assays are crucial to eliminate false positive “hits” as well as evaluate their biological functions. A rational workflow was established for screening PD-1/PD-L1 inhibitors (Fig. 5a). SPR technology was performed to evaluate binding affinities between small molecule and PD-L1. The identified PD-L1 inhibitors were selected for PD-1/PD-L1 pair ELISA assay. Once the inhibitors exert blockage effects on PD-1/PD-L1 interactions, bioluminescence reporter cell-based assay can be applied for determining their biological functions. The identified PD-L1 inhibitor without blockage effects on PD-1/PD-L1 interactions is a "false" positive hit. For instance, punicalagin (PA) is an ellagitannin found in pomegranate (*Punica granatum*). Our screening data demonstrated that PA exhibits a stronger binding affinity with PD-L1 than BMS-1166, a positive PD-L1 inhibitor (Fig. 4b). The K_D_ value of 5.5 × 10^–10^ M is determined by SPR. Notably, the PD-1/PD-L1 pair ELISA demonstrated that PA only showed minor blockage effects against PD-1/PD-L1 interactions (Fig. 5b). As discussed, biophysical methods, such as SPR, provides binding parameters of identified inhibitor with PD-1 and/or PD-L1. However, PA might not exert blockage effects towards PD-1/PD-L1 even if it has strong binding affinities with PD-L1. Future studies with in vivo models are warranted to confirm this.

As summarized, the PD-1/PD-L1 interface is challenging to target because of its large and flat hydrophobic interface. Binding parameters need to be measured for the small molecules and PD-1/PD-L1 interactions including the ones at their interactive interface and other non-interactive sites. Therefore, it is important to properly apply complementary approaches including biophysical, biochemical, and cell-based assays to achieve robust measurements. These combination strategies are critical to eliminate false positive “hits” (such as PA as demonstrated in this review), which may only have binding capacity without blockage effects on PD-1/PD-L1 interaction. Nevertheless, we believe that more versatile and advanced bioassays can be developed in the future to shed more light on the discovery of PD-1/PD-L1 inhibitors.

## Data Availability

Not applicable.

## Abbreviations

APCs: Antigen-presenting cells
BLI: Biolayer interferometry
CD: Circular dichroism
CLTA-4: Cytotoxic T-lymphocyte-associated protein 4
DCs: Dendritic cells
DSF: Differential scanning fluorometry
ELISA: Enzyme-linked immunosorbent assay
FPIA: Fluorescence polarization immunoassay
HTRF: Homogeneous time resolved fluorescence
irAEs: Immune-related adverse effects
IFN: Interferon
Ig: Immunoglobulin
IL: Interleukin
ITC: Isothermal titration calorimetry
ITIM: Immunoreceptor tyrosine-based inhibitory motif
ITSM: Immunoreceptor tyrosine-based switch motif
MDS: Microfluidic diffusional sizing
MST: Microscale thermophoresis
mTOR: Mechanistic targets of rapamycin
NMR: Nuclear magnetic resonance
PA: Punicalagin
PCR: Polymerase chain reaction
PD-1: Programmed cell death protein 1
PD-L1: Programmed death-ligand 1
PI3K: Phosphatidylinositol 3-kinase
SHP: Src homology 2 domain-containing protein tyrosine phosphatase
SPE: *Salvia plebeia* R. Br. Extract
SPR: Surface plasmon resonance
TCR: T-cell receptor
w-AIDA-NMR: Weak-antagonist induced dissociation assay-NMR
WB: Western blotting
